# Changes in the Microbiome Profile in Different Parts of the Intestine in Piglets with Diarrhea

**DOI:** 10.3390/ani12030320

**Published:** 2022-01-28

**Authors:** Mariya V. Gryaznova, Yuliya D. Dvoretskaya, Mikhail Y. Syromyatnikov, Sergey V. Shabunin, Pavel A. Parshin, Evgeniy V. Mikhaylov, Nikolay A. Strelnikov, Vasily N. Popov

**Affiliations:** 1 Laboratory of Metagenomics and Food Biotechnology, Voronezh State University of Engineering Technologies, 394036 Voronezh, Russia; mariya-vg@mail.ru (M.V.G.); dyd16@mail.ru (Y.D.D.); pvn@vsuet.ru (V.N.P.); 2 Department of Genetics, Cytology and Bioengineering, Voronezh State University, 394018 Voronezh, Russia; 3 FSBSI All-Russian Veterinary Research Institute of Pathology, Pharmacology and Therapy, 394061 Voronezh, Russia; svshabunin@rambler.ru (S.V.S.); doktor.57@mail.ru (P.A.P.); voronezh81@rambler.ru (E.V.M.); strelnickov.nickolay@yandex.ru (N.A.S.)

**Keywords:** piglet, microbiome, 16S rRNA, sequencing, diarrhea, intestines

## Abstract

**Simple Summary:**

The most common genera in the piglet microbiome were *Lactobacillus*, *Escherichia-Shigella*, *Enterococcus*, *Bacteroides*, and *Fusobacterium*. Bacteria of the *Lactobacillus* genus dominated in healthy piglets. An increased number of *Escherichia-Shigella* and *Enterococcus* was detected in diarrheal pigs. This indicates an important role of these bacteria in the pathogenesis of diarrhea. A decreased number of *Bacteroides* was detected in diarrheal pigs. According to the assessment of the microbiome composition in different sections of the intestine, bacteria of the *Lactobacillus* genus were the most common in the ileum, while *Fusobacterium* and *Bacteroides* were more common in the rectum. Our results show that the gut microbiome may make a significant contribution to the pathogenesis of diarrhea.

**Abstract:**

Determining the taxonomic composition of microbial consortia of the piglet intestine is of great importance for pig production. However, knowledge on the variety of the intestinal microbiome in newborn piglets is limited. Piglet diarrhea is a serious gastrointestinal disease with a high morbidity and mortality that causes great economic damage to the pig industry. In this study, we investigated the microbiome of various sections of the piglet intestine and compared the microbiome composition of healthy and diarrheal piglets using high-throughput sequencing of the 16S rRNA gene. The results showed that bacteria of the *Lactobacillus* genus were the most common in the ileum, while *Fusobacterium* and *Bacteroides* dominated in the rectum. Comparing the microbiome composition of healthy and diarrheal piglets revealed a reduced number of *Lactobacillus* bacteria as a hallmark of diarrhea, as did an increased content of representatives of the *Escherichia-Shigella* genus and a reduced number of *Bacteroides*, which indicates the contribution of these bacteria to the development of diarrhea in piglets. The relative abundance of *Enterococcus* bacteria was higher in the diarrhea group. Although some bacteria of this genus are commensals, a small number of species may be associated with the development of diarrhea in piglets. Therefore, our results indicate that the gut microbiome may be an important factor in the development of diarrhea in piglets.

## 1. Introduction

The formation of the gut microbiome at an early age is of particular importance for piglets’ health. Microbiome composition is a perspective predictive tool for health and disease assessment; however, it stays poorly described in terms of predisposition to diarrhea. Here, we aim to assess whether the composition of the gut microbiome is associated with differences in the susceptibility of pigs to diarrhea.

The gut microbiome has a complex organization, characterized by multiple options for inter-bacterial interaction, which greatly contributes to the health and immunity of mammals [1]. It is characterized by a large population diversity of microorganisms, the composition and abundance of which are influenced by both external and internal factors, such as environmental factors and the genetics of the host organism [2].

Populations of intestinal bacteria are essential in animal production. The development of high-throughput sequencing technologies has significantly advanced the study of the microbiomes of production animals [3,4].

The trend in recent years shows an exponential increase in the number of publications using the 16S rRNA gene sequencing approach to study the gut microbiome of pigs [5,6,7,8]. Interestingly, the composition of the pig gut microbiome correlates with such indicators as average body weight and daily weight gain [9,10], conversion factor, and food consumption [11,12]. At the same time, quite a small number of studies are devoted to a detailed analysis of microbiome profiles in different parts of the intestines of piglets [13,14,15]. In addition, only pigs over 120 days old or weaned pigs were analyzed in those studies. The dynamics of gut microbiome formation in newborn piglets are of particular importance as it affects the overall animal health and growth indicators [16].

It is known that from birth the digestive system of piglets is populated with facultative aerobic or anaerobic bacteria. They receive this compound together with colostrum and then from breast milk, which contains lactic acid bacteria such as *Lactobacilli* and *Bifidobacterium* as the main probiotic bacteria. The gut microbiome composition of piglets then changes and stabilizes, influenced by feeding and environmental characteristics [17,18]. The data show that the intestines of piglets are mainly colonized by the *Clostdiaceae* and *Enterobacteriaceae* families immediately after birth. One of the studies showed that the *Streptococcaceae* family can be observed in the gastrointestinal tract of piglets 6 h after birth, and in the period from 1 to 3 days it becomes the most numerous. Then, as a result of secondary colonization, they are gradually replaced by *Lactobacillaceae* and *Clostridiaceae* [19]. Other studies show the attendance of bacterial species such as *Lactobacillus sobrius*, *Escherichia coli*, *Lactobacillus reuteri*, *Shigella flexneri*, and *Lactobacillus acidophilus* in two-day-old pigs [17]. *E. coli* and *Clostridium* spp. were observed during the first 6 h after birth. *Bacteroidetes* spp. appear four days after birth and are therefore considered the latest representative of the emerging microbiota [20]. According to a study by Petri et al. [19], *Lactobacillaceae* were the most considerable part during the first 20 days of life, which contradicts the results, according to which representatives of this group are replaced by *Clostridium* spp. [20,21]. According to some studies, the microbiological composition of the digestive tract of piglets is quite stable during the lactation period [22,23,24,25]. Thus, the diversity of available data indicates the demand to investigate issues related to the bacterial composition of different parts of the intestine from the birth of piglets. 

Pigs and piglets suffer from a variety of infections that can be caused by the environment, food, internal parasites, bacteria, viruses, and/or the simultaneous effect of these factors. These infections are a serious threat to agronomic health and ultimately to human health [26,27]. Hence, studying infectious conditions in pigs can help not only to improve the health of livestock but also to develop new treatments against bacterial infections. Diarrhea is considered to be one of the major microbial diseases in domestic piglets, and can have devastating consequences for animal health and therefore the food industry [28]. Post-weaning diarrhea is an economically important intestinal disease because of the financial losses that it causes [29]. It commonly occurs within 2 weeks after weaning and is characterized by profuse diarrhea, dehydration, high mortality (up to 20–30% within 1–2 months [29]), and weight loss in surviving pigs [30].

The main aim of this investigation was to assess the differences between the intestine microbiomes of piglets with diarrhea and healthy animals. We also studied the association between the composition of the intestinal bacterial community and the susceptibility of piglets to diarrhea. For this purpose, we studied the composition of the microbiome in four sections of the piglet intestine (ileum, cecum, colon, and rectum) using 16S rRNA gene sequencing on the Ion Torrent Personal Genome Machine (PGM) platform.

## 2. Materials and Methods

### 2.1. Samples

Twelve 3- to 4-day-old piglets (F1 hybrids) were obtained by crossing animals of the Large White and Landrace breeds. Six piglets with severe diarrhea and six healthy piglets were selected for the experiment and kept in a room at 30 ± 2 °C with a humidity of 55 ± 7%. Piglets with diarrhea were identified by the following clinical picture: shaky, unstable gait against the background of general dehydration, loose stools, fetid smell of fecal masses, and general contamination of piglets with feces. Molecular analysis of pathological material (feces, internal organs from piglets (thin and thick intestine, liver with gallbladder, kidney, and spleen)) did not detect viral diseases. Microscopy of smears-prints from the intestinal surface and in the feces did not detect pathogens of parasitic diseases. The piglets were euthanized and portions of their gastrointestinal tract were immediately removed. The contents of the intestinal lumen were collected from the ileum, cecum, colon, and rectum. In total, 48 samples were obtained (24 from sick and 24 from healthy animals), which were placed in microcentrifuge tubes and delivered to the laboratory on ice. However, in the course of sample preparation, seven samples of the gastrointestinal tract taken from the sick group of piglets were removed from the subsequent analysis, including one sample of the ileum, three of the cecum, one of the colon, and two of the rectum. Thus, a total of 41 samples of the gastrointestinal tract of pigs were sequenced: in the sick group, 17 samples were examined, of which five were of the ileum, three were of the cecum, five were of the colon, and four were of the rectum; in the healthy group, 24 samples were examined—six from each section of the intestine.

### 2.2. Isolation of DNA

DNA was extracted from each sample using a ZymoBiomics DNA Miniprep Kit (Zymo Research, Los Angeles, CA, USA) according to the manufacturer’s instructions. The isolated DNA was quantified using a Qubit 2.0 benchtop fluorometer (Invitrogen, San Diego, CA, USA).

During DNA isolation from samples, we added the sample that served as a negative control for data analysis and contained only the Milli-Q water which is used in the laboratory. This sample underwent sample preparation completely identically to the test samples to exclude the contamination of the test samples in the laboratory. During the bioinformatics analysis of the obtained data we used the decontam R package to identify and subsequently remove any laboratory contaminants. This additional step makes it possible to obtain more accurate data on the composition of the studied bacterial communities, which is based on the analysis of the V3 hypervariable region of the 16S rRNA marker gene and metagenomic data.

### 2.3. Amplification of the 16S rRNA Gene

In our work, to study the gut microbiome of piglets using sequencing on the Ion Torrent PGM platform we selected the hypervariable region V3 of the 16S rRNA gene. For the amplification of bacterial DNA we used universal primers 337F and 518R (Table 1).

The amplification was performed with a 5 × ScreenMix-HS Master Mix kit (Evrogen, Moscow, Russia) in the following regime: 94 °C for 4 min; 37 cycles of 94 °C for 30 s, 53 °C for 30 s, and 72 °C for 30 s; and final elongation at 72 °C for 5 min.

### 2.4. Ion Torrent PGM Sequencing

PCR products were purified using AMPureXP magnetic particles (Beckman Coulter, Brea, CA, USA). The preparation of libraries for sequencing was performed using a NEBNext Fast DNA Library Prep kit (New England Biolabs, Ipswich, MA, USA) according to the manufacturer’s protocol. After that, the obtained libraries were mixed in equimolar amounts for emulsion PCR on a OneTouch 2 System (Thermo Fisher Scientific, Madison, WI, USA). Sequencing was performed with an Ion PGM Hi-Q View Sequencing Kit (Thermo Fisher Scientific, Madison, WI, USA) using an Ion Torrent PGM system.

### 2.5. Statistical Data Analysis

The sets of sequences for each sample were obtained in a BAM format. Next, the files were converted into a FastQ format using SAMtools v.1.2 software and analyzed using the R programming language in the RStudio environment. The raw reads were filtered by length and the quality was controlled by using the functions of VSEARCH v.2.8.2 software. The samples were pooled and unique sequences were identified before searching for operational taxonomic units (OTUs). To find the OTUs, we used the UNOISE2 algorithm, which reduces noise by correcting errors. To generate the OTUs and make the OTU table, we combined all the reads for all the samples. After that, the processed reads were consistent with the reference readings in the SILVA v.123 databases (https://www.arb-silva.de, accessed on 26 October 2021) using the DADA2 package. The DADA2 package provides a native implementation of the naive Bayesian classifier method for this purpose. 

The statistical analysis was performed using GraphPad Prism 9 software (GraphPad, Sand Diego, CA, USA). The differences in the bacterial composition of the microbiomes from the intestine sections were analyzed using two-way analysis of variance (ANOVA). The results are expressed as mean ± standard error of the mean (SEM).

## 3. Results

**Figure 1:**
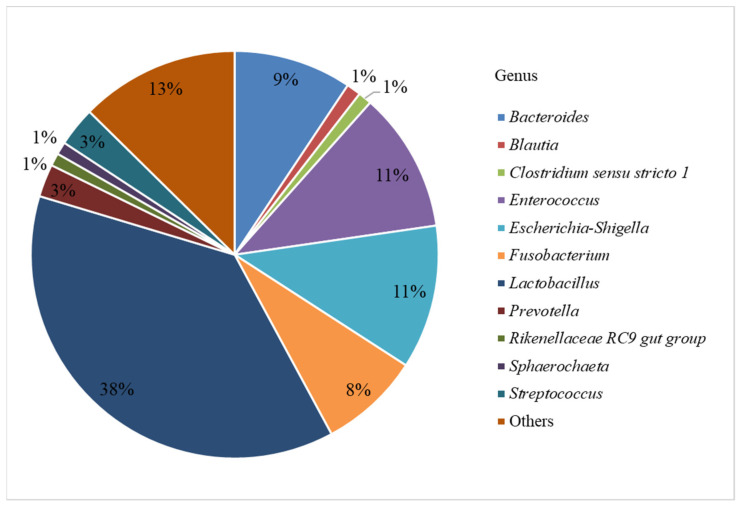
Relative abundance of identified bacterial genera.

In this study, we investigated the bacterial profiles (at the level of genera) of the ileum, cecum, colon, and rectum from twelve piglets. After filtering the reads obtained by sequencing of 41 studied samples, a total of 307,977 unique sequences were identified, which corresponded to 166 genera (99% identity) (Figure 1).

Figure 1 shows that 38% of the sequences belonged to members of the *Lactobacillus* genus. The next in numbers were *Escherichia-Shigella* and *Enterococcus* (11% each), *Bacteroides* (9%), *Fusobacterium* (8%), *Streptococcus* and *Prevotella* (3% each), and *Blautia*, *Clostridium sensu stricto 1*, *Rikenellaceae RC9* gut group, and *Sphaerochaeta* (1% each). The genera with the content below 1% were combined into the “Others” group.

**Figure 2:**
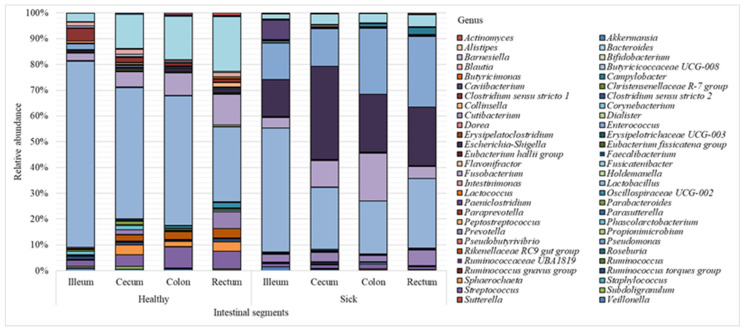
Microbiome composition (bacterial genera) of piglet intestine.

The results revealed the differences between the microbial profiles of different sections of piglet gastrointestinal tract, as well as between sick and healthy piglets (the 40 most common genera identified for each intestinal section are shown in Figure 2). The bacterial composition of individual samples can be found in Supplementary Material Figure S1.

Figure 2 shows that the *Lactobacillus* genus prevailed over other genera in all sections of the intestine; however, a greater number of these bacteria was found in healthy piglets (51% on average) vs. animals with diarrhea (30% on average). Bacteria of the *Escherichia*-*Shigella* genus were also found in large numbers (24%) in sick piglets compared to 1% in healthy animals. Representatives of the *Bacteroides* genus were more common in the healthy group (14%) vs. 4% in sick piglets. The abundance of *Streptococcus* in the healthy animals was 6%, while in piglets with diarrhea, the content of these bacteria was approximately 1%. In the healthy group, the amount of *Sphaerochaeta* was 2%; in the sick group, these bacteria were absent. The relative content of representatives of the *Enterococcus* genus in the healthy and sick groups was 1% and 20%, respectively.

**Figure 3:**
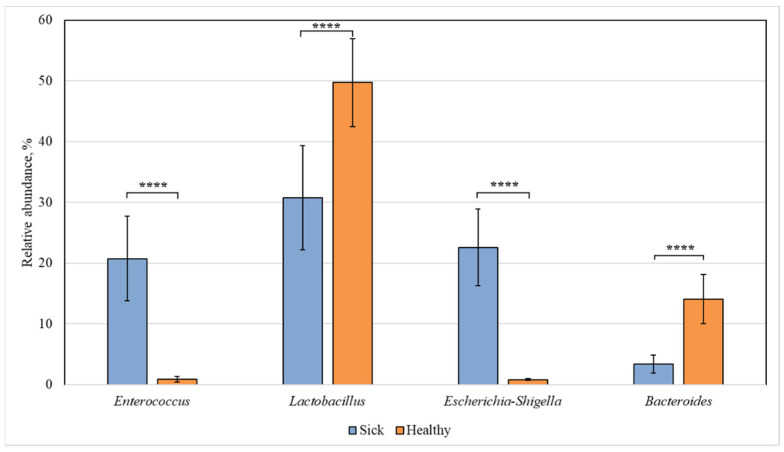
Statistically significant differences in the content of bacterial genera in the intestine of healthy and diseased piglets. Results are expressed as mean ± SEM (**** *p* ≤ 0.0001).

Figure 3 shows the bacterial genera, the intestinal content of which differed statistically in the healthy and diseased groups.

Among all identified bacterial genera, statistically significant differences were found for the representatives of *Enterococcus*, *Lactobacillus*, *Escherichia-Shigella*, and *Bacteroides*. The content of *Enterococcus* and *Escherichia-Shigella* bacteria in the sick group increased (to 21 and 23%, respectively) compared to the healthy animals, in which their relative abundance was 1% for both genera. At the same time, in sick piglets, the content of *Lactobacillus* (31%) and *Bacteroides* (3%) was reduced compared to 50% and 14%, respectively, in the healthy group.

**Figure 4:**
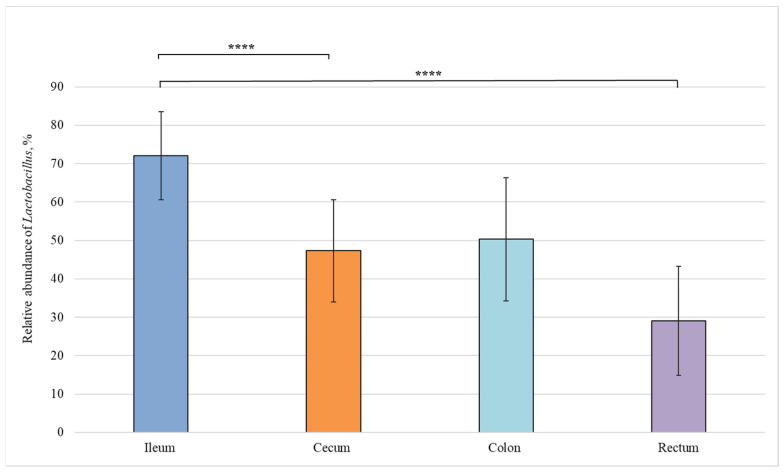
Relative abundance of *Lactobacillus* genus in the intestinal sections of healthy piglets. Results are expressed as mean ± SEM (**** *p* ≤ 0.0001).

**Figure 5:**
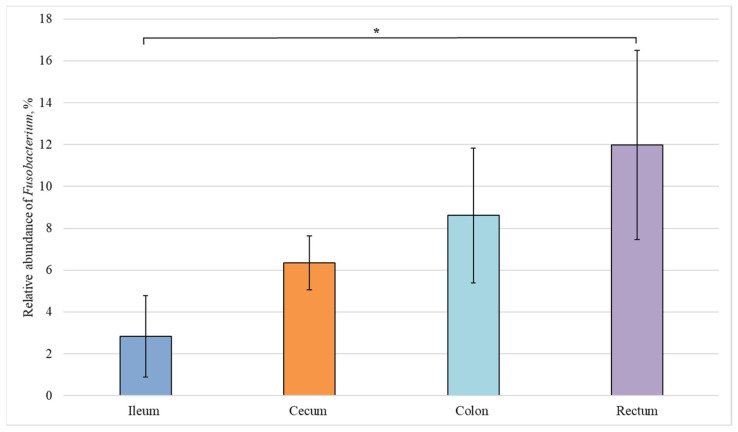
Relative abundance of the *Fusobacterium* genus in the intestinal sections of healthy piglets. Results are expressed as mean ± SEM (* *p* ≤ 0.05).

**Figure 6:**
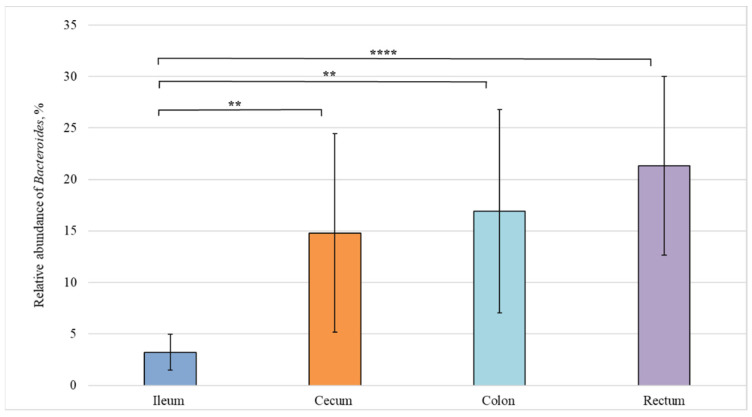
Relative abundance of the *Bacteroides* genus in the intestinal sections of healthy piglets. Results are expressed as mean ± SEM (** *p* ≤ 0.01, **** *p* ≤ 0.0001).

We also carried out a comparative analysis of the bacterial composition in different sections of the intestine from healthy piglets and revealed statistically significant differences in the relative abundance of bacteria belonging to the *Lactobacillus*, *Fusobacterium*, and *Bacteroides* genera (Figure 4, Figure 5 and Figure 6).

Figure 4 shows that the amount of *Lactobacillus* bacteria in the ileum (72%) was significantly higher than in the cecum (50%) and rectum (29%).

The relative number of representatives of the *Fusobacterium* genus increases in the direction from the small to the large intestine (Figure 5). However, a statistically significant difference was observed only between the content of *Fusobacterium* bacteria in the ileum and rectum (3% and 12%, respectively), i.e., in the most distant sections.

Figure 6 demonstrates a statistically significant difference in the content of *Bacteroides* genus between the ileum (3%) and cecum (15%), as well as between the ileum (3%) and colon (21%).

**Figure 7:**
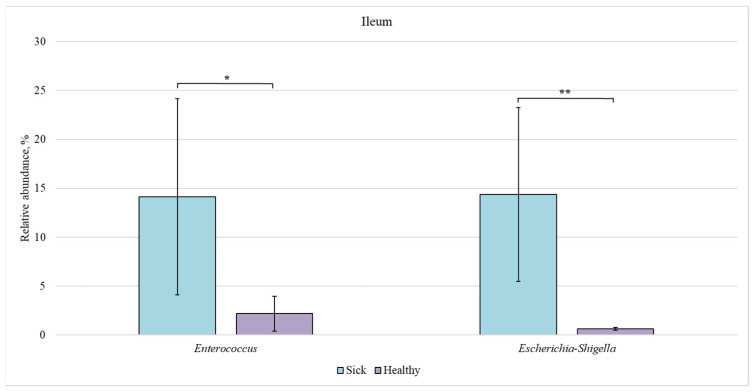
Relative abundance of *Enterococcus* and *Escherichia-Shigella* bacteria in the ileum of sick and healthy piglets. Results are expressed as mean ± SEM (* *p* ≤ 0.05, ** *p* ≤ 0.01).

**Figure 8:**
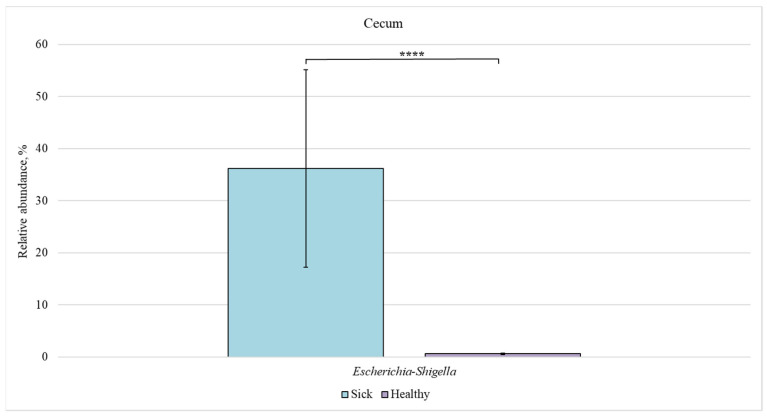
Relative abundance of *Escherichia-Shigella* bacteria in the cecum of sick and healthy piglets. Results are expressed as mean ± SEM (**** *p* ≤ 0.0001).

**Figure 9:**
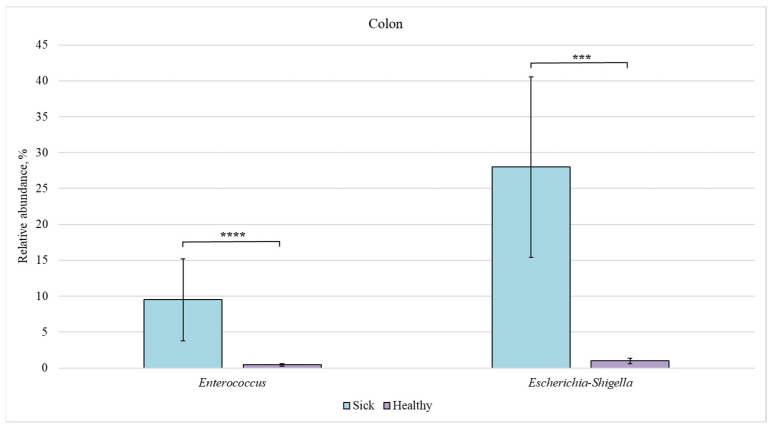
Relative abundance of *Enterococcus* and *Escherichia-Shigella* bacteria in the colon of sick and healthy piglets. Results are expressed as mean ± SEM (*** *p* ≤ 0.001, **** *p* ≤ 0.0001).

**Figure 10:**
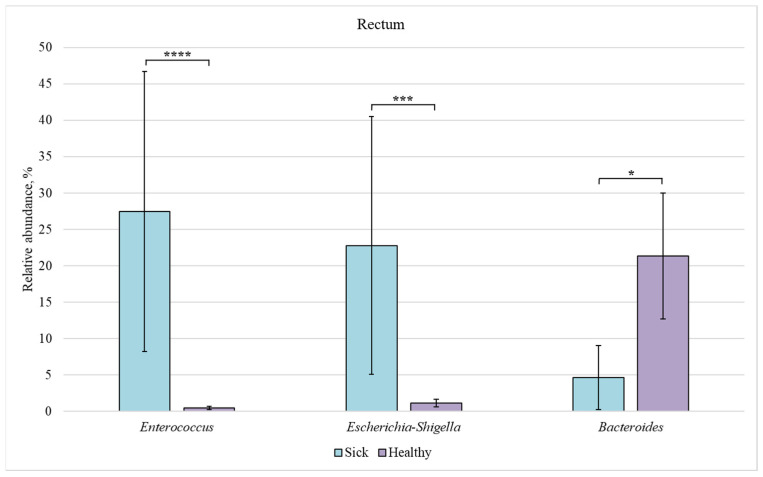
Relative abundance of *Enterococcus*, *Escherichia-Shigella*, and *Bacteroides* bacteria in the rectum of sick and healthy piglets. Results are expressed as mean ± SEM (* *p* ≤ 0.05, *** *p* ≤ 0.001, **** *p* ≤ 0.0001).

Next, we compared the bacterial composition of each intestinal section in the healthy and sick groups (Figure 7, Figure 8, Figure 9 and Figure 10).

In the ileum of sick piglets, the content of *Enterococcus* and *Escherichia-Shigella* bacteria (14% each) was approximately 7 and 20 times higher than in the healthy animals (2 and 0.7%, respectively).

In the cecum, a significant difference in the relative content was found only for the *Escherichia-Shigella* genus (36% in sick animals vs. 0.6% in healthy piglets).

The relative content of *Enterococcus* (9.5%) and *Escherichia-Shigella* (28%) bacteria was increased in the colon and ileum of sick piglets (compared to 0.5% and 1%, respectively, in the healthy group).

In the rectum, a significant difference in the relative abundance was observed for the three genera. The content of *Enterococcus* and *Escherichia-Shigella* bacteria was increased in the sick group as compared to the healthy animals (27% and 23% vs. 0.5% and 1%, respectively). However, the number of representatives of the *Bacteroides* genus was reduced in sick piglets (5%) in comparison with the healthy ones (21%).

## 4. Discussion

In this study, we discovered that *Lactobacillus* was one of the major genera of the pig gastrointestinal tract (Figure 1). *Lactobacillus* representatives are common in both proximal and distal sections of pig digestive tract, which they colonize shortly after birth [31].

*Lactobacillus* bacteria possess several probiotic properties necessary for resistance to infections and diseases of the gastrointestinal tract. They exhibit antipathogenic activity [32,33] and antioxidant activity [34,35] and are involved in the regulation of immune system [36]. All these traits affect gut microbial populations, such that propagation of opportunistic pathogens, such as *Salmonella*, *Clostridia*, and *Enterobacteriaceae*, is controlled, resulting in the prevention of infections and intestinal disorders [37,38,39]. It has been shown that *Lactobacillus* species colonize piglet intestine shortly after birth and are stable members of the gut microbiome throughout the entire intestinal tract [40]. The low abundance of *Lactobacillus* representatives, which are considered beneficial, may be an indicator of gastrointestinal problems in pigs. In our study (Figure 3), we observed a reduced amount of *Lactobacillus* bacteria in piglets with diarrhea, which confirms the probiotic properties of the *Lactobacillus* genus.

Physiological variations along the length of the intestine include chemical and nutrient gradients, as well as compartmentalized host immune activity. All these factors influence bacterial community composition [41]. It is well known that piglets experience extreme stress when they are weaned from the sow. This can lead to the development of intestinal dysfunction and immune system disorders, which ultimately leads to a deterioration in the health of piglets, especially during the first week after weaning. We would like to point out that only suckling piglets were involved in this study. This made it possible to exclude the influence of stress endured during weaning on changes in the microbiome and, as a consequence, the development of diarrhea [42].

According to the results of our study, *Lactobacillus* dominated in the distal part of the small intestine (ileum) (Figure 4). *Lactobacillus* species grow in an oxygen-free or low-oxygen atmosphere. Oxygen present in the small intestine is gradually depleted by aerobic bacteria, and only a small amount of it remains in the ileum, where most *Lactobacillus* bacteria are found. Some *Lactobacillus* species produce acetic acid, which has a fairly strong antipathogenic effect [43].

In our study, the content of *Escherichia*-*Shigella* bacteria was much higher in piglets with diarrhea (Figure 3). Several *Escherichia-Shigella* species are believed to play an important role in the development of diarrhea in piglets and to have a serious effect on the barrier function of the animal intestine [44]. An increased number of *Escherichia-Shigella* bacteria in sick piglets vs. healthy ones was observed in all sections of the intestine (Figure 7, Figure 8, Figure 9 and Figure 10). However, we found no differences in the number of these bacteria in the intestine sections in the healthy group. This suggests that *Escherichia-Shigella* bacteria are distributed approximately evenly in the investigated sections of the intestine.

Members of the *Bacteroides* genus are often found in the digestive tract of mammals. They are early colonizers of the intestines of healthy piglets [19]. *Bacteroides* species play an important role in health promotion by producing butyrate, which activates T cell-mediated immune response, thus limiting colonization of the digestive tract by potentially pathogenic bacteria [45]. In the healthy group, bacteria of this genus were more common in the rectum (Figure 6). The number of *Bacteroides* representatives in the rectum of piglets with diarrhea was lower than in the control group (Figure 10).

We also found an increased number of *Enterococcus* bacteria in sick piglets (Figure 3). Information on the enterococcal flora of healthy newborn piglets is scanty, but a small number of representatives of this genus can be a part of the normal gut microbiota [46,47]. However, *Enterococcus* is sometimes associated with piglet diarrhea [48,49]. Although many members of the *Enterococcus* genus are believed to be commensals of the intestinal tract, some representatives cause diarrhea in suckling animals of various species [50,51,52,53,54,55].

We also found an increased number of *Enterococcus* bacteria in the ileum (Figure 7), colon (Figure 9), and rectum (Figure 10) of piglets with diarrhea compared to healthy animals. This may indicate that pathogenic *Enterococcus* representatives are prevalent in the intestinal microbiome, which may be associated with the diarrhea of newborns piglets.

*Fusobacterium* species are anaerobic, Gram-negative, non-spore-forming, non-motile, rod-shaped bacteria. The main metabolite produced by these bacteria is butyric acid [56]. According to numerous data, *Fusobacterium* is considered to be a normal representative of the oropharyngeal, gastrointestinal and genital microbiota. At the same time, this genus is the second most commonly isolated anaerobic microbial group from clinical samples of both humans and animals, especially in the case of purulent-necrotic infections [57]. *Fusobacterium* is involved in various clinical anaerobic infections and can cause intestinal inflammation [58]. Even though many studies have shown increased levels of *Fusobacterium* in piglets with various intestinal disorders compared to the healthy ones [59,60,61,62,63], we did not find statistically significant differences in the content of this genus in the studied groups. However, we found an increased content of *Fusobacterium* members in the rectum compared to the ileum of healthy pigs. The content of *Fusobacterium* bacteria in newborn piglets requires further research.

We studied the bacterial composition inhabiting the intestines of healthy newborn piglets, as well as piglets with diarrhea. Thus, our study, using high-throughput sequencing, showed that microbial communities in the studied samples included many commensal and opportunistic microorganisms. Compared to classical microbiological and immunological approaches, as well as PCR, the advantage of this method is the ability to identify all bacteria contained in the test sample, including non-culturable microorganisms [64]. We used the universal primer pair 337F/518R (see Section 2.3. Amplification of the 16S rRNA gene) to amplify a fragment of the 16S rRNA gene that includes the V3 region, which is considered one of the most effective hypervariable regions for phylogenetic analysis and taxonomic classification of bacterial species [65,66].

The results of this study can be used to predict bacterial taxa indicative of healthy development of gut microbiome in suckling piglets, as well as to identify taxa that can be used as probiotics to prevent post-weaning diarrhea.

## 5. Conclusions

The most common genera in the piglet microbiome were *Lactobacillus*, *Escherichia-Shigella*, *Enterococcus*, *Bacteroides*, and *Fusobacterium*. Bacteria of the *Lactobacillus* genus dominated in healthy piglets, which once again proves their probiotic effect. An increased number of *Escherichia-Shigella* representatives in diarrheal pigs indicates the contribution of these bacteria to the development of diarrhea. A decreased number of *Bacteroides* may also indicate the development of diarrhea. The content of *Enterococcus* bacteria was higher in sick piglets. According to the assessment of the microbiome composition in different sections of the intestine, bacteria of the *Lactobacillus* genus were most common in the ileum, while *Fusobacterium* and *Bacteroides* were more common in the rectum.

Further studies are needed to understand the protective mechanisms of the gut microbial community and to develop clinical interventions to improve gut health in piglets.

## Figures and Tables

**Table 1:** Primers used in the study.

Primer	Sequence
337F	5′-GACTCCTACGGGAGGCWGCAG-3′
518R	5′-GTATTACCGCGGCTGCTGG-3′

## Data Availability

Raw sequencing data are available in the NCBI BioProject database (BioProject ID: PRJNA794980).

## Supplementary Materials

The following supporting information can be downloaded at: https://www.mdpi.com/article/10.3390/ani12030320/s1, Figure S1: The abundance of microorganisms for the samples.
