# Supplementary figures and images for: Changes in the Microbiome Profile in Different Parts of the Intestine in Piglets with Diarrhea
Source: Animals (Basel). 2022 Jan 28;12(3):320. doi: 10.3390/ani12030320 (PMC8833389; doi:10.3390/ani12030320)

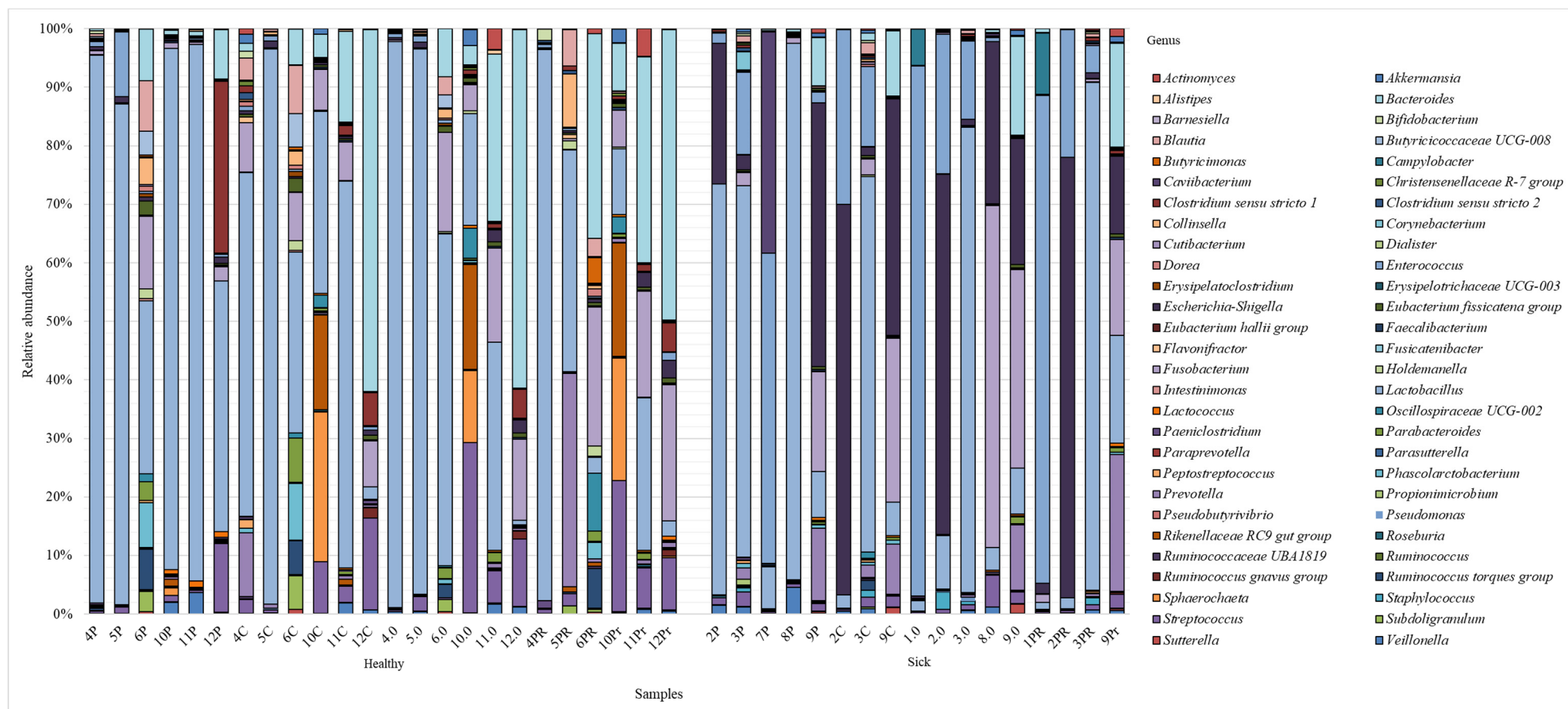

**Figure S1.** The abundance of microorganisms for the samples.

Supplement: Supplementary file 1 [file animals-12-00320-s001.zip › animals-1522132-supplementary.pdf]
